# Pediatric lymphatic leishmaniasis: a case report

**DOI:** 10.1186/s13256-023-03852-x

**Published:** 2023-04-06

**Authors:** Endeshaw Asaye Kindie, Ermias Teklehaimanot Yefter, Bewketu Abebe Alemu, Tiruzer Bekele Gurji, Amanuel Kassa Tadesse

**Affiliations:** Department of Pathology, University of Gondar College of Medicine and Health Sciences, Gondar, Ethiopia

**Keywords:** Leishmaniasis, Lymph node, *Leishmania donovani* bodies

## Abstract

**Background:**

There are three main forms of leishmaniases: visceral (the most serious form because it is almost always fatal without treatment), cutaneous (the most common, usually causing skin ulcers), and mucocutaneous (affecting mouth, nose, and throat). Leishmaniasis is caused by protozoan parasites, which are transmitted by the bite of infected female phlebotomine sandflies. The disease affects some of the world’s poorest people and is associated with malnutrition, population displacement, poor housing, a weak immune system, and lack of financial resources. An estimated 700,000 to 1 million new cases occur annually. Only a small fraction of those infected by parasites causing leishmaniasis will eventually develop the disease. We report a case of exclusive lymph node involvement in leishmaniasis, presenting as localized lymphadenopathies. The diagnosis of lymphatic leishmaniasis was confirmed by the presence of *Leishmania donovani* bodies in fine needle aspiration cytology, and positive anti-rK39 antibodies. The bone marrow aspiration was negative for *Leishmania donovani* bodies. Abdominal ultrasound was done and there was no organomegaly. Furthermore, localized lymphadenopathies may provide a diagnostic challenge by clinically mimicking a lymphoma or other causes of lymphadenopathy. Due to its rarity and its tendency to pose a clinical diagnostic challenge, we decided to report a case of lymphatic leishmaniasis.

**Case presentation:**

A 12-year-old Amara male patient presented to the University of Gondar comprehensive specialized hospital, Northwestern Ethiopia, with six discrete right lateral cervical lymphadenopathies, the largest measuring 3 × 2 cm^2^, with no cutaneous lesion. Fine needle aspiration cytology confirmed the diagnosis of leishmaniasis in lymph node, and he was put on sodium stibogluconate (20 mg/kg body weight/day) and paromomycin (15 mg/kg body weight/day) injections, which are given intramuscularly for 17 days. Having completed his medication at the University of Gondar comprehensive specialized hospital, he had a smooth course and was discharged with appointment scheduled for follow-up after 3 months.

**Conclusion:**

In the clinical evaluation of a patient with isolated lymphadenopathies, leishmaniasis must be considered as a differential diagnosis in immunocompetent subjects in endemic areas for early diagnostic workup and management.

## Background

Leishmaniasis consists of a group of diverse diseases that affect viscera, skin, and/or mucous membranes, with a wide spectrum of clinical activity caused by vector-borne, obligate, intracellular hemoflagellates of the genus *Leishmania*. Three major clinical syndromes are recognized: visceral, cutaneous, and mucocutaneous leishmaniasis. The clinical manifestations of leishmaniasis appears to depend on a complex set of factors, including tropism and virulence of the parasite strain, and the susceptibility of the host, which may be genetically determined [1].

Visceral leishmaniasis (VL), also known as kala-azar, is fatal if left untreated in over 95% of cases. It is characterized by irregular bouts of fever, weight loss, enlargement of the spleen and liver, and anemia. Most cases occur in Brazil, East Africa, and India. An estimated 50,000 to 90,000 new cases of VL occur worldwide annually, with only 25–45% reported to World Health Organization. It has outbreak and mortality potential. Cutaneous leishmaniasis (CL) is the most common form and causes skin lesions, mainly ulcers, on exposed parts of the body. These can leave lifelong scars and cause serious disability or stigma. About 95% of CL cases occur in the Americas, the Mediterranean Basin, the Middle East, and Central Asia. It is estimated that 600,000 to 1 million new cases occur worldwide annually, but only around 200,000 are reported to WHO. Mucocutaneous leishmaniasis leads to partial or total destruction of mucous membranes of the nose, mouth, and throat. Over 90% of mucocutaneous leishmaniasis cases occur in Bolivia (the Plurinational State of Bolivia), Brazil, Ethiopia, and Peru [2]. In Ethiopia, the annual VL infection rate is about 4500, of which 10–20% is coinfected with human immunodeficiency virus (HIV). HIV-infected people are particularly vulnerable to VL, while VL accelerates HIV’s progression to acquired immune deficiency syndrome (AIDS) [3]. According to “Leishmaniasis in Ethiopia: A systematic review and meta-analysis of prevalence in animals and humans,” the overall random pooled prevalence of leishmaniasis was 19% [95% confidence interval (CI) 14–24%] [4].

The most important clinical manifestation of visceral leishmaniasis is the syndrome known as kala-azar (Hindi for “black fever”). The incubation period is usually 2–6 months, but can range from a few weeks to several years. Onset of symptoms is usually insidious or sub-acute, with slow progression of malaise, fever, weight loss, and splenomegaly (with or without hepatomegaly) over a period of months [5]. *Leishmania* invade and replicate within host macrophages, evading innate and cell-mediated immune responses. Infection generally appears to persist after clinical cure of the primary infection [6, 7]. Evasion and persistence are achieved through a combination of strategies, including neutralization of complement components, preventing release of macrophage superoxide and nitric oxide, and suppressing induction of antigen-specific CD4^+^ T-helper lymphocytes [7, 8].

Localized leishmanial lymphadenopathies (LLL) are lymphadenopathies (> 1 cm cervical or axillary, > 2 cm inguinal) at the first clinical manifestation, with parasitologic confirmation by stained smear, culture, or polymerase chain reaction (PCR) from lymph node biopsy or aspirated material [9]. Definitive diagnosis of leishmaniasis requires the demonstration of parasite by smear or culture in tissue (usually bone marrow or spleen). The utility of less invasive diagnostic tools (such as demonstration of specific antibodies, antigens, or parasite DNA in peripheral blood specimens) depends on the clinical status of the patient, the geographic origin of the parasite, the methods employed, and laboratory experience [10].

Herein, we report a case of exclusive involvement of lymph node in leishmaniasis, presenting as localized lymphadenopathies in the right lateral cervical lymph node, which was clinically considered as a lymphoma. Additionally, as it is evident in our case presentation, the condition can pose a diagnostic challenge for clinicians by resembling lymphoma or other causes of lymphadenopathy.

As a result, we are reporting this case of uncommon occurrence, due to its rarity, as a seat of origin in this anatomic location and to emphasize the importance of considering leishmaniasis in the differential diagnosis of isolated lymphadenopathies in immunocompetent subjects in endemic areas.

## Case presentation

**Fig. 1:**
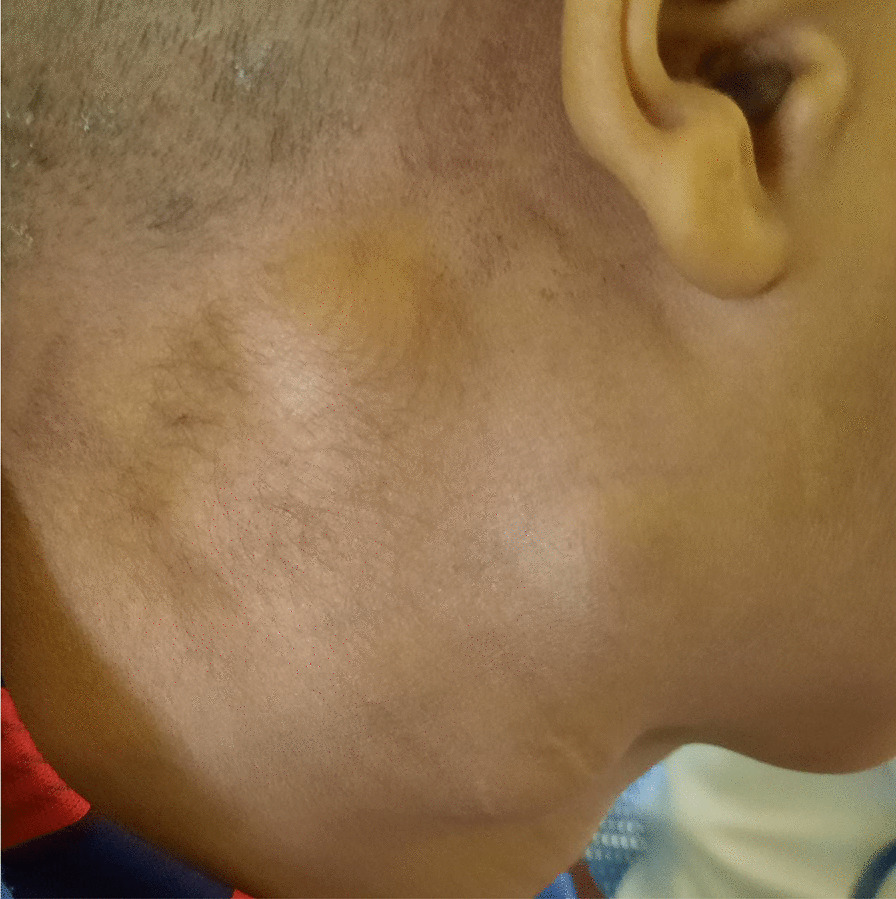
A 12-year-old Amara male patient presented with localized leishmanial lymphadenopathies with no cutaneous lesion

A 12-year-old Amara male patient presented to University of Gondar comprehensive specialized hospital, Northwestern Ethiopia, with a complaint of right lateral neck swelling of 6 months duration. The swellings were painless and reported to have increased in size. He had visited a nearby health center on multiple occasions for this complaint and took unspecified antibiotics, but no improvement. There was no history of fever, cough, weight loss, night sweating, loss of appetite, pressure effects, or bowel, bladder, joints or nervous system involvement. He had no family history (first-degree relatives) of diabetes, hypertension, or any other remarkable noncommunicable disease, including cancer. His past medical history is not significant. He had no history of admission to hospital. He had no history of any form of surgical procedures. He was a grade four student in a public school at the time of presentation. On physical examination, there were six discrete firm, nontender, freely mobile right lateral cervical lymphadenopathies, the largest measuring 3 × 2 cm^2^ (Fig. 1). On the basis of the above findings, a provisional clinical impression of lateral cervical lymphadenopathy, due to either lymphoma or tuberculosis, was entertained.

Liver was not palpable below costal margin. There was no splenomegaly. Other clinical findings were within normal limits. Laboratory investigations done on the same day of his presentation, including complete blood count (CBC), erythrocyte sedimentation rate (ESR), and chest X-ray, were noncontributory. On CBC, total white blood cell (WBC) count was 5800 µL with 50% granulocytes, 45% lymphocytes, 1% eosinophils, and 4% monocytes. Platelet count was 300,000 µL. Hemoglobin was 14.5 g/dL with mean corpuscular volume (MCV) of 88 fL. ESR was 14 mm/hour. Renal function test revealed blood urea nitrogen (BUN) of 12 mg/dL, and serum creatinine level was 0.68 mg/dL. On liver function test, total bilirubin was 0.6 mg/dL, serum albumin was 4.2 g/dL, and serum aspartate transaminase (AST/SGOT) and serum alanine transaminase (ALT/SGPT) were 30 and 32 IU/L, respectively. Urinalysis was also done and it was normal. Sputum was negative for acid-fast bacilli. Serum was negative for HIV antibody. Chest X-ray and ultrasound of abdomen did not reveal any abnormality.

**Fig. 2:**
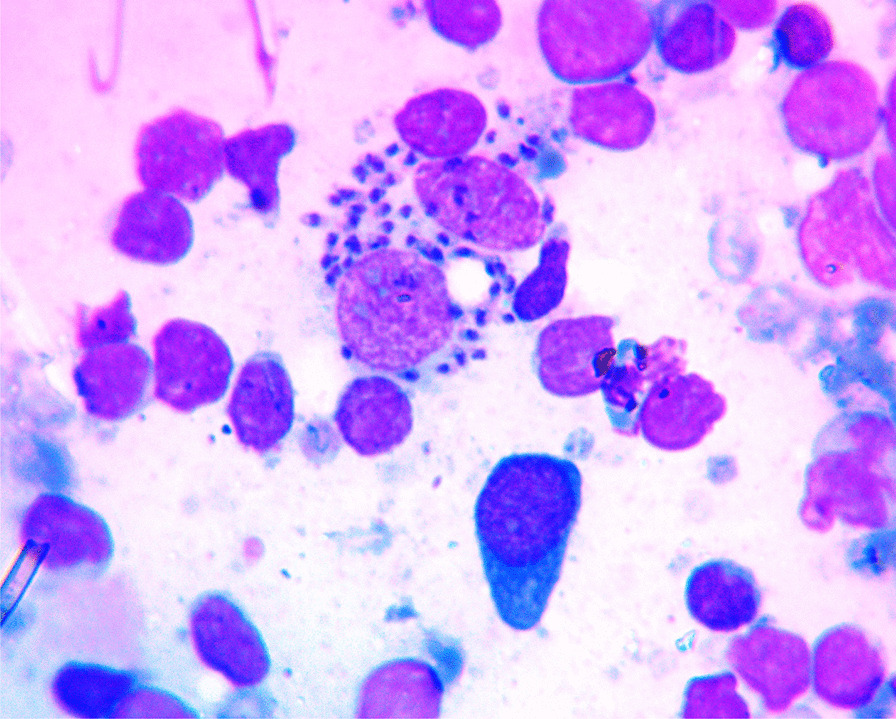
Fine needle aspiration cytology smear of lymph node showing intracellular *Leishmania donovani* bodies (Wright’s stain)

**Fig. 3:**
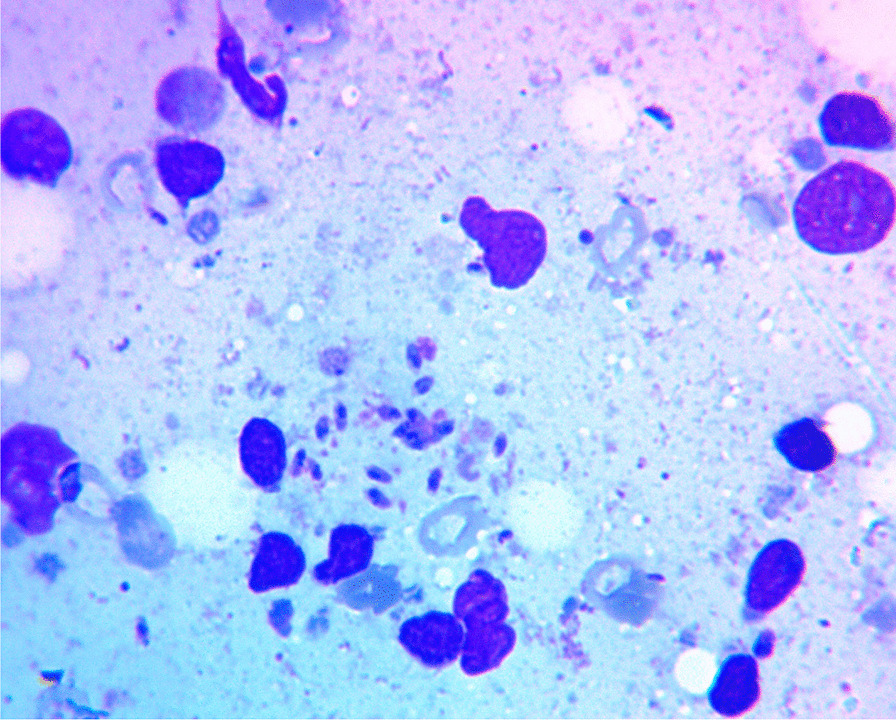
Fine needle aspiration cytology smear of lymph node showing occasional extracellular *Leishmania donovani* bodies (Wright’s stain)

Due to limited number of pathologists and long waiting list of patients, he underwent fine needle aspiration cytology (FNAC). After 2 weeks of his initial presentation, FNAC from cervical lymph node was done and smears were stained with Wright’s stain following the standard procedures. Microscopic examination showed intracellular and extracellular *Leishmania donovani* (LD) bodies on polymorphous background (Figs. 2, 3). Serum tested for anti-rK39 antibodies was strongly positive. Bone marrow aspirations done on two occasions were negative for LD bodies. On the basis of above findings, the case was diagnosed as lymphatic leishmaniasis and the patient was put on sodium stibogluconate (20 mg/kg body weight/day), and paromomycin (15 mg/kg body weight/day) injections, which were given intramuscularly for 17 days and defined as the preferred first-line regimens for primary VL in Ethiopia.

Having completed his medication at the University of Gondar comprehensive specialized hospital, he had a smooth course and was discharged with an appointment scheduled for follow-up after 3 months. When he came for the follow-up after 3 months, he had right lateral neck small mobile lymph nodes and was discharged with advice to return to the University of Gondar comprehensive specialized hospital if there was any new-onset increment of his right lateral neck small mobile lymph nodes.

## Discussion

Due to the preference of *Leishmania* parasites for the monocyte–macrophage system, generalized lymphadenopathy can occur with classical visceral leishmaniasis [11]. Lymph node enlargement associated with cutaneous lesions caused by *Leishmania tropica* or by *Leishmania major* has been reported [12]. In the New World, lymphadenopathy may accompany, or even precede, skin lesions due to the *Leishmania braziliensis* complex, indicating that spread from the skin to lymph nodes occurs early in the course of infection [13, 14]. However, localized lymphadenopathies without cutaneous or systemic manifestations are unusual and have rarely been reported [14, 15]. Most cases were diagnosed by surgical biopsy because of a suspected lymphoma. Interestingly, in most of these patients, the lymph node enlargement affected the left neck without further specification. In our case, the affected lymph node was located on the right neck.

Ethiopian leishmaniasis is usually not accompanied by lymph node enlargement, although manifestation of leishmaniasis in the form of superficial lymph node enlargement, without any visceral involvement, is known to occur in Mediterranean countries, Africa, and China [16]. Similar to our case, the majority of patients with lymphatic leishmaniasis do not have a fever. Three distinct natural courses of LLL have been described. Some of these patients remain as LLL, some develop full-blown kala-azar, and others develop dermal lesions [16, 17]. In a series of atypical presentations in adults in the largest community outbreak of leishmaniasis in Europe (Fuenlabrada, Spain), there were 90 adults with LLL or VL who were treated; 72% were men, and the mean age was 46.2 years (range 15–95 years). A total of 17 cases (19%) were LLL, an atypical form with isolated lymphadenopathies without other symptoms. All LLL cases occurred in immunocompetent subjects, and only one subject (6%) was a native of sub-Saharan Africa. Similar to our case, a total of 73 subjects (81%) presented with typical VL; 66% of this group were immunocompetent, and 50% of those who were immunocompetent were descendants of natives of sub-Saharan Africa. The rK39 test and polymerase chain reaction were the most useful tests for confirmation of the diagnosis. An initial response to treatment was observed in 99% of cases, and relapses occurred in 14% of cases. LLL appeared as long evolution lymphadenopathies (median 60 days, interquartile range 75), and no patient presented with either fever or any other systemic symptom during the evolution. Nine cases (53%) had more than one lymphadenopathy, with two (12%) in different lymphadenopathy areas. The most frequent location was the cervical area (11 cases, 65%), including the laterocervical, submandibular, and parotid chains. Our case also presented with right lateral cervical lymphadenopathy (LAP). Six patients (35%) presented with inguinal lymphadenopathies, three patients (18%) presented with axillary lymphadenopathies, and one case presented with supraclavicaular and costal lymphadenopathies. Unlike our case, six patients (35%) presented with a cutaneous lesion, suspicious of cutaneous leishmaniasis, before the appearance of the lymphadenopathy. Diagnosis was performed in all LLL cases by fine needle aspiration cytology of the lymphadenopathy.

The same was true for our case. The pathologic finding was nonnecrotizing granulomatous lymphadenitis with *Leishmania* parasites in all cases, except for one that did not exhibit granulomas. Serology was negative in 38%. Serum tested for anti-rK39 antibodies was strongly positive for our case. There has only been one recorded patient with LLL who had clinical and radiologic splenomegaly, and anemia, leukopenia, and thrombocytopenia [18]. Like our case, four cases of British Servicemen serving in Malta and Cyprus have been described, in whom the only clinical abnormality was the presence of enlarged lymph nodes, and in three of whom LD bodies were demonstrated in material obtained by lymph node biopsy or aspiration [19]. Tissues from ten patients with LLL were stained by immunoperoxidase technique for Leishmanian antigens, immunoglobulins (Ig), complement, components, α_1_-antitrypsin, and fibrin and by routine and chloroacetate esterase techniques. Lymph nodes with *Leishmania* lymphadenitis showed a granulomatous process with a varying degree of necrosis, Leishmanian amastigotes, abundant plasma cells, and focal fibrosis [20]. Our case FNAC smear of lymph node also showed intracellular and extracellular *Leishmania donovani* bodies on polymorphus background.

The significance of LLL lies in the fact that the majority of cases remain asymptomatic, and therefore do not seek medical advice until much later. Thus, they remain undiagnosed and serve as a potential source of parasite in the community. On the other hand, the diagnosis is often overlooked in view of other common causes of generalized lymphadenopathy, such as tuberculosis, lymphoma, leprosy, and fungal infection. In our patient, who reported from endemic area of kala-azar, the localized leishmanial lymphadenopathies, in the absence of other significant clinical and laboratory findings, initially misled the clinician. Lack of suspicion of lymphatic leishmaniasis led to a long period of suffering for the patient. The patient and his parents were very satisfied with the intervention and care given.

## Conclusion

Localized lymph node enlargement due to infection with *Leishmania* needs to be included in the spectrum of unusual presentations of leishmanial infections, and localized leishmanial lymphadenopathies as a differential diagnosis need to be kept in mind when a patient with localized lymphadenopathies is encountered from an area endemic for kala-azar.

## Data Availability

Data sharing does not apply to this article as no new data were created or analyzed in this study.

## Abbreviations

WHO: World Health Organization
ALT: Alanine transaminase
AST: Aspartate transaminase
BUN: Blood urea nitrogen
CBC: Complete blood count
ESR: Erythrocyte sedimentation rate
FNAC: Fine-needle aspiration cytology
MCV: Mean corpuscular volume
WBC: White blood cell
LD body: *Leishmania donovani* bodies
LLL: Localized leishmanial lymphadenopathies
VL: Visceral leishmaniasis
PCR: Polymerase chain reaction
LAP: Lymphadenopathy
CL: Cutaneous leishmaniasis
